# MicroRNA-148a Targets *DNMT1* and *PPARGC1A* to Regulate the Viability, Proliferation, and Milk Fat Synthesis of Ovine Mammary Epithelial Cells

**DOI:** 10.3390/ijms25168558

**Published:** 2024-08-06

**Authors:** Jiqing Wang, Na Ke, Xinmiao Wu, Huimin Zhen, Jiang Hu, Xiu Liu, Shaobin Li, Fangfang Zhao, Mingna Li, Bingang Shi, Zhidong Zhao, Chunyan Ren, Zhiyun Hao

**Affiliations:** Gansu Key Laboratory of Herbivorous Animal Biotechnology, College of Animal Science and Technology, Gansu Agricultural University, Lanzhou 730070, China; wangjq@gsau.edu.cn (J.W.); ken@st.gsau.edu.cn (N.K.); wuxinmiao2020@163.com (X.W.); zhenhm@st.gsau.edu.cn (H.Z.); huj@gsau.edu.cn (J.H.); liuxiu@gsau.edu.cn (X.L.); lisb@gsau.edu.cn (S.L.); zhaofangfang@gsau.edu.cn (F.Z.); limn@gsau.edu.cn (M.L.); shibg@gsau.edu.cn (B.S.); zhaozd@gsau.edu.cn (Z.Z.); renyaya86@126.com (C.R.)

**Keywords:** microRNA-148a, ovine mammary epithelial cells, viability, proliferation, triglyceride, *DNMT1*, *PPARGC1A*

## Abstract

In this study, the expression profiles of miR-148a were constructed in eight different ovine tissues, including mammary gland tissue, during six different developmental periods. The effect of miR-148a on the viability, proliferation, and milk fat synthesis of ovine mammary epithelial cells (OMECs) was investigated, and the target relationship of miR-148a with two predicted target genes was verified. The expression of miR-148a exhibited obvious tissue-specific and temporal-specific patterns. miR-148a was expressed in all eight ovine tissues investigated, with the highest expression level in mammary gland tissue (*p* < 0.05). Additionally, miR-148a was expressed in ovine mammary gland tissue during each of the six developmental periods studied, with its highest level at peak lactation (*p* < 0.05). The overexpression of miR-148a increased the viability of OMECs, the number and percentage of Edu-labeled positive OMECs, and the expression levels of two cell-proliferation marker genes. miR-148a also increased the percentage of OMECs in the S phase. In contrast, transfection with an miR-148a inhibitor produced the opposite effect compared to the miR-148a mimic. These results indicate that miR-148a promotes the viability and proliferation of OMECs in Small-tailed Han sheep. The miR-148a mimic increased the triglyceride content by 37.78% (*p* < 0.01) and the expression levels of three milk fat synthesis marker genes in OMECs. However, the miR-148a inhibitor reduced the triglyceride level by 87.11% (*p* < 0.01). These results suggest that miR-148a promotes milk fat synthesis in OMECs. The dual-luciferase reporter assay showed that miR-148a reduced the luciferase activities of DNA methyltransferase 1 (*DNMT1*) and peroxisome proliferator-activated receptor gamma coactivator 1-A (*PPARGC1A*) in wild-type vectors, suggesting that they are target genes of miR-148a. The expression of miR-148a was highly negatively correlated with *PPARGC1A* (r = −0.789, *p* < 0.001) in ovine mammary gland tissue, while it had a moderate negative correlation with *DNMT1* (r = −0.515, *p* = 0.029). This is the first study to reveal the molecular mechanisms of miR-148a underlying the viability, proliferation, and milk fat synthesis of OMECs in sheep.

## 1. Introduction

Milk is a nutrient substance synthesized and secreted by mammalian mammary gland tissue. Ovarian hormones are responsible for mammary gland growth and the process of milk secretion. For example, estrogen contributes to the growth of secretory ducts, while progesterone affects the differentiation and development of lactiferous vesicles. Prolactin, oxytocin, glucocorticoids, growth hormone, and locally secreted growth factors are also involved in the lactation process. The importance of milk yield and nutrients in milk to the dairy sheep industry is evident. In non-dairy sheep, these factors are also vital for the survival rate, pre-weaning growth rate, and development of lambs, especially in multiple-born lambs breeds. Because mammary epithelial cells (MECs) are the basic structural unit of lactation, their viability and proliferation primarily determine the milk yield and composition in dairy animals. In this context, MECs have become research models and hotspots for investigating lactation performance and milk composition. If regulatory factors or biological mechanisms underlying the activities of MECs can be revealed, opportunities to genetically improve lactation performance may arise. 

It is well known that the viability and proliferation of MECs are regulated by many functional genes and non-coding RNAs, including microRNAs (miRNAs). miRNAs are endogenous non-coding RNAs ranging from 18 to 25 nucleotides in length. Most miRNAs down-regulate the expression of target mRNAs by binding complementally to their 3’-untranslated regions (3’ UTR). Although miRNAs account for less than 5% of the mammalian genome, they can regulate the expression of up to 60% of functional genes [1]. These observations suggest that miRNAs are important post-transcriptional regulatory factors and play essential roles in many biological processes. To date, research studies on the effects of miRNAs on MECs or lactation traits have mainly focused on dairy goats and dairy cows. Studies have reported the effects of miRNAs on milk fat synthesis in MECs and revealed that miRNAs are responsible for milk fat synthesis. For example, miR-193a-5p was found to target fatty acid desaturase 1 (*FADS1*) to inhibit the content of polyunsaturated fatty acid in dairy cows [2]. miR-26a and miR-26b increased the levels of unsaturated fatty acid and triacylglycerol, and the expression of genes associated with fatty acid synthesis [3], suggesting a positive regulatory role in milk fat synthesis. The effect of miR-106b [4], miR-16a [5], and miR-128 [6] on milk fat metabolism has also been documented. However, there are few studies on the effect of miRNAs on milk protein. miR-204-5p and miR-211 targeted casein alpha _S1_ (*CSN1S1*) to inhibit its content but enhanced the level of casein beta (CSN2) in dairy goats [7].

While the importance of miRNAs in lactation performance and mammary gland development has been well established in some mammals, there are very few reports on the effect of single miRNAs on mammary epithelial cells (OMECs) in sheep. Wang et al. [8] found that miR-199a-3p increased the viability and proliferation of OMECs, but inhibited milk fat synthesis by targeting the very low-density lipoprotein receptor gene (*VLDLR*). miR-200c was found to increase triglyceride levels by targeting the pantothenate kinase 3 gene (*PANK3*). In contrast, miR-432 targeted the stearoyl-CoA desaturase gene (*SCD*) and lipoprotein lipase gene (*LPL*) to inhibit milk fat synthesis in OMECs [9]. 

In a previous study on ovine mammary glands, miR-148a was identified as one of the most highly expressed miRNAs, both at peak lactation and during the non-lactating period [1]. miR-148a was the most up-regulated miRNA at peak lactation compared with the non-lactating period [1]. These observations suggest that miR-148a may be involved in the regulation of mammary gland development and lactation performance in sheep. We hypothesized that miR-148a regulates ovine lactation traits by targeting specific genes. To test this hypothesis, we detected the expression levels of miR-148a in eight different ovine tissues, including mammary gland tissue, during six different development periods. We also analyzed the effect of miR-148a on the viability, proliferation, and milk fat synthesis of OMECs, and we verified its target relationship with the DNA methyltransferase 1 gene (*DNMT1*) and the peroxisome proliferator-activated receptor gamma coactivator 1-A gene (*PPARGC1A*). The results reveal the biological mechanism by which miR-148a regulates mammary gland development and lactation performance in sheep. The aim of this study was to lay a theoretical foundation for improving the lactation performance of sheep.

## 2. Results

### 2.1. Tissue Expression of miR-148a

**Figure 1:**
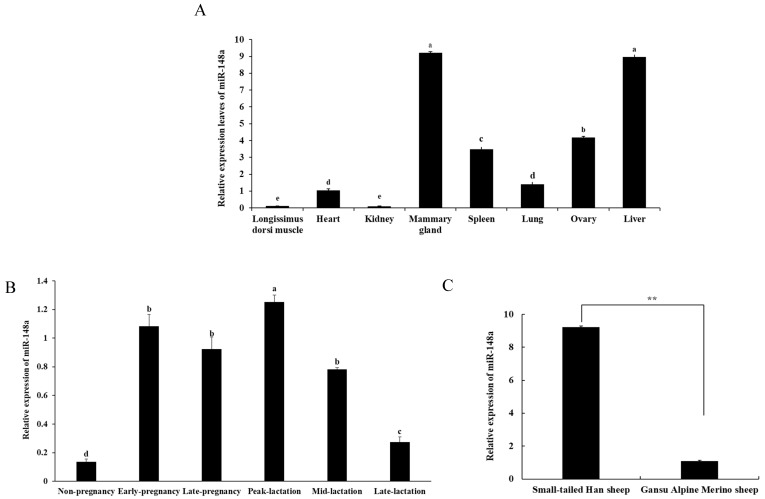
Expression levels of miR-148a in ovine eight different tissues (**A**), mammary gland tissue during different developmental periods (**B**) and mammary gland tissue at peak lactation of Small-tailed Han sheep and Gansu Alpine Merino sheep (**C**). Values with different lowercase letters above the bars are different (*p* < 0.05). ** *p* < 0.01.

The RT-qPCR analysis of the eight different tissues collected from Small-tailed Han sheep revealed that miR-148a was not only expressed in the mammary gland, but also in all of the other seven tissues, including the *Longissimus dorsi* muscle, heart, kidney, spleen, lung, ovary, and liver. miR-148a had the highest expression level in the mammary gland and liver, while it was expressed at the lowest level in the *Longissimus dorsi* muscle and kidney (Figure 1A, *p* < 0.05). Moreover, miR-148a was widely expressed in the mammary gland tissue across six different development periods, but it exhibited a temporal-specific expression pattern. Specifically, the expression of miR-148a was the highest at peak lactation, while it was the lowest in late lactation and during the non-pregnancy periods. The expression level of miR-148a at peak lactation was 9.24-fold, 1.16-fold, 1.35-fold, 1.60-fold, and 4.57-fold higher than in non-pregnancy, early pregnancy, late pregnancy, mid-lactation, and late lactation, respectively (Figure 1B, *p* < 0.05). It was notable that in the mammary gland at peak lactation, the expression of miR-148a was 8.48-fold higher in Small-tailed Han sheep compared to Gansu Alpine Merino sheep which have lower milk yield and lower milk fat and milk protein contents (Figure 1C, *p* < 0.01). These results suggest that miR-148 may be involved in the regulation of mammary gland development and lactation performance in sheep.

### 2.2. miR-148a Increases the Viability of OMECs

**Figure 2:**
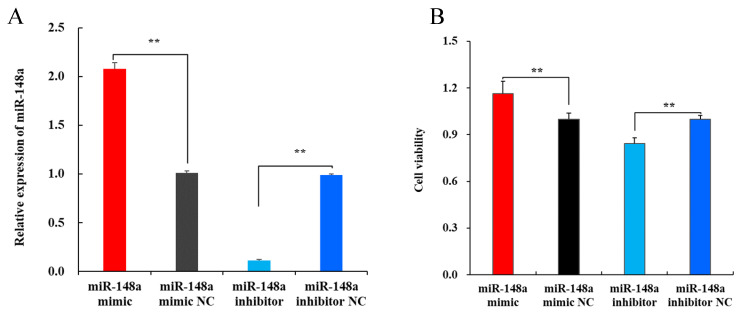
Transfection efficiency of miR-148a detected using RT-qPCR (**A**) and its effect on the viability of ovine mammary epithelial cells (**B**). ** *p* < 0.01.

The transfection efficiency of the miR-148a mimic and miR-148a inhibitor into OMECs was validated by RT-qPCR analysis. The results revealed that the transfection of the miR-148a mimic into OMECs significantly increased the expression of miR-148a in the cells compared to the miR-148a mimic NC (Figure 2A, *p* < 0.01). In contrast, the expression level of miR-148a in OMECs transfected with the miR-148a inhibitor decreased to 11.48% of that in the miR-148a inhibitor NC (Figure 2A, *p* < 0.01). These results suggest that the miR-148a mimic and miR-148a inhibitor were successfully transfected into OMECs in the study.

The CCK8 analysis revealed that the miR-148a mimic led to a significant increase in the viability of OMECs compared to the miR-148a mimic NC group (Figure 2B, *p* < 0.01), while the viability was markedly decreased in OMECs transfected with the miR-148a inhibitor when compared to its NC group (Figure 2B, *p* < 0.01).

### 2.3. miR-148a Improves the Proliferation of OMECs

**Figure 3:**
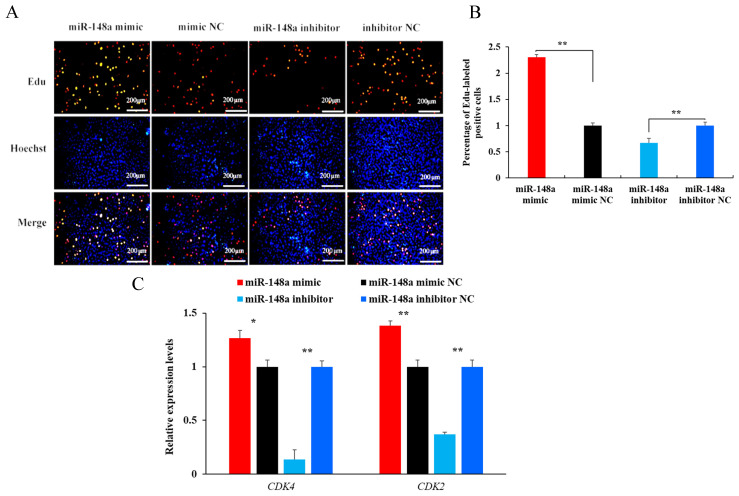
Effect of miR-148a on the proliferation of ovine mammary epithelial cells (OMECs) when the miR-148a mimic and miR-148a inhibitor were transfected into OMECs. (**A**) Proliferation of OMECs detected using an Edu assay. (**B**) Percentage of Edu-labeled positive OMECs. (**C**) Relative expression levels of *CDK4* and *CDK2*. ** *p* < 0.01 and * *p* < 0.05.

The effect of miR-148a on the proliferation of OMECs was investigated by counting the number and percentage of Edu-labeled positive OMECs and by detecting the expression of the cell proliferation marker genes cyclin-dependent kinase 2 (*CDK2*) and *CDK4*. The Edu assay showed that the miR-148a mimic significantly increased the number of Edu-labeled positive OMECs (Figure 3A). Conversely, the miR-148a inhibitor produced the opposite effect compared to the miR-148a mimic (Figure 3A). Statistical analysis revealed that the percentage of Edu-labeled positive OMECs was 2.30-fold higher in the miR-148a mimic group than in the NC group (Figure 3B, *p* < 0.01), while the miR-148a inhibitor decreased the percentage (Figure 3B, *p* < 0.01). 

As shown in Figure 3C, the miR-148a mimic increased the expression of *CDK2* and *CDK4* in OMECs (*p* < 0.01), while the miR-148a inhibitor resulted in decreased expression of the genes (*p* < 0.01). Taken together, these results suggest that miR-148a improves the proliferation of OMECs.

### 2.4. miR-148a Affects the Cycle of OMECs

**Figure 4:**
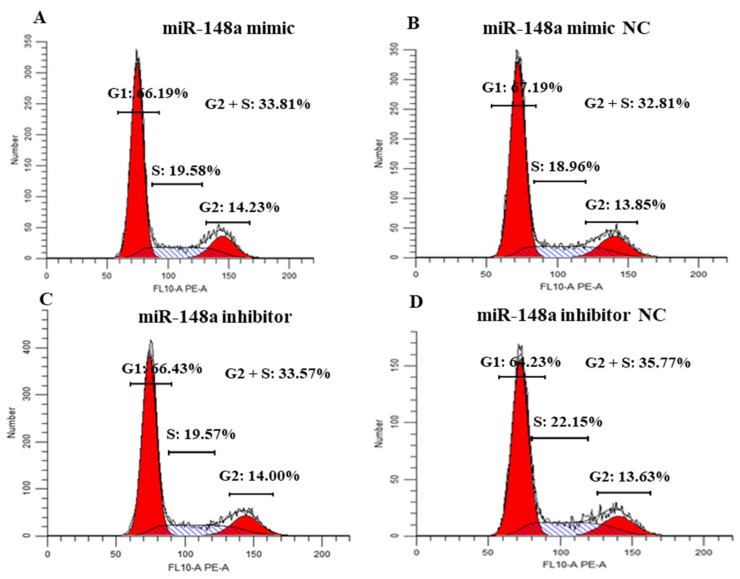
Effects of the miR-148a mimic (**A**) and miR-148a inhibitor (**C**) on the cycle of ovine mammary epithelial cells (OMECs) when compared to the miR-148a mimic NC (**B**) and miR-148a inhibitor NC (**D**).

Flow cytometry analysis revealed that the miR-148a mimic promoted the transition of OMECs from the G1 phase to the S phase when compared to the miR-148a mimic NC group (Figure 4A,B). In contrast, the percentage of OMECs in the S phase was decreased by 2.58% in the miR-148a inhibitor group compared to its NC group (Figure 4C,D).

### 2.5. miR-148a Promotes Milk Fat Synthesis

**Figure 5:**
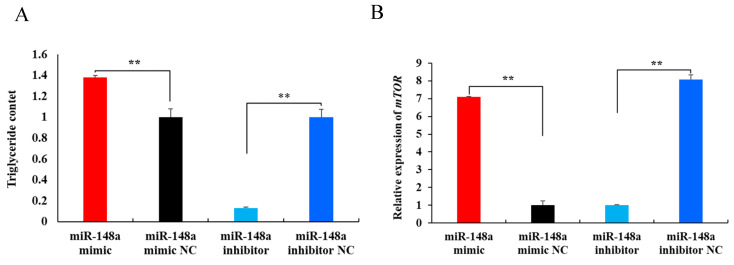

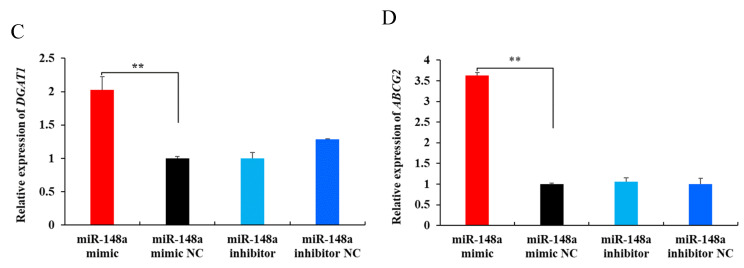
Effect of miR-148a on the triglyceride level (**A**) and expression levels of *mTOR* (**B**), *DGAT1* (**C**), and *ABCG2* (**D**) in ovine mammary epithelial cells (OMECs). ** *p* < 0.01.

Triglyceride level detection revealed that the miR-148a mimic increased the triglyceride content by 37.78% compared to the miR-148a mimic NC (*p* < 0.01). In contrast, the miR-148a inhibitor reduced the triglyceride content by 87.11% (Figure 5A, *p* < 0.01). 

To further verify the effect of miR-148a on milk fat synthesis in OMECs, the expression levels of three marker genes were assessed using RT-qPCR analysis. As shown in Figure 5B–D, the miR-148a mimic increased the expression of mechanistic target of rapamycin (*mTOR*), diacylglycerol acyltransferase 1 (*DGAT1*), and ATP binding cassette subfamily G member 2 (*ABCG2*) (*p* < 0.01), while the miR-148a inhibitor decreased the expression of *mTOR* (*p* < 0.01). In summary, these results indicate that miR-148a promotes milk fat synthesis in OMECs in sheep. 

### 2.6. miR-148a Targets DNMT1 and PPARGC1A

**Figure 6:**
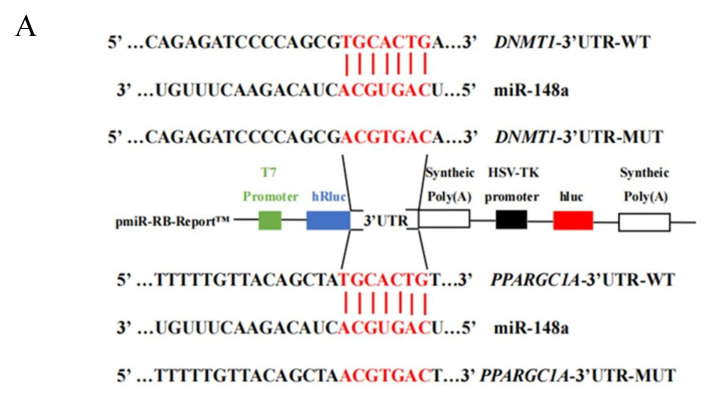

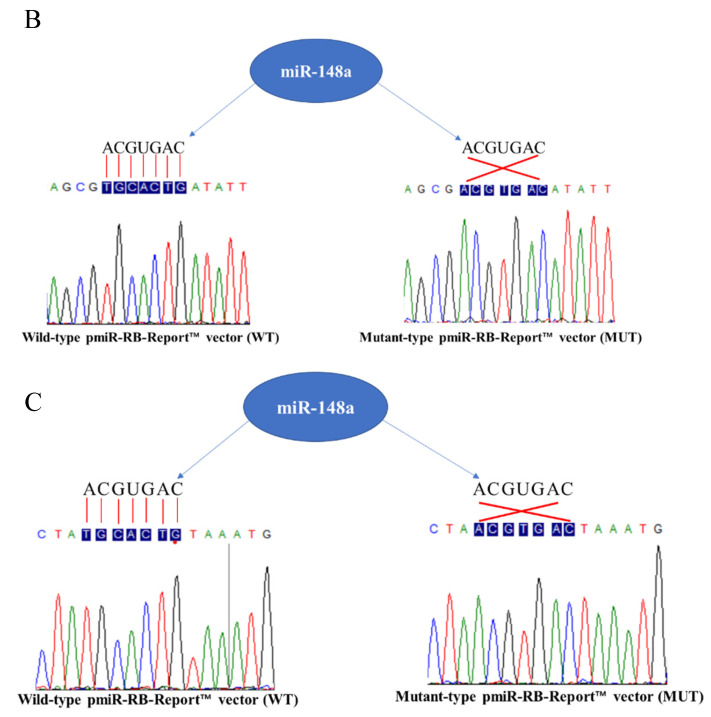
Construction and sequencing results of dual luciferase reporter vectors for two target genes. (**A**) The structural diagram of dual luciferase reporter vectors. (**B**) Sequence validation of the target gene *DNMT1* in wild-type (WT) and mutant-type (MUT) pmiR-RB-Report™ vectors by Sanger sequencing. (**C**) Sequence validation of the target gene *PPARGC1A* in WT and MUT pmiR-RB-Report™ vectors by Sanger sequencing.

After the joint prediction of miRDB, TargetScan7.2, and miRWalk, miR-148a was found to target *DNMT1* and *PPARGC1A*. To validate this target relationship, dual luciferase reporter vectors were designed (Figure 6A). Sanger sequencing results confirmed the presence of the expected sequences in both the wild-type and mutant-type pmiR-RB-Report™ vectors (Figure 6B,C), indicating successful vector construction. 

**Figure 7:**
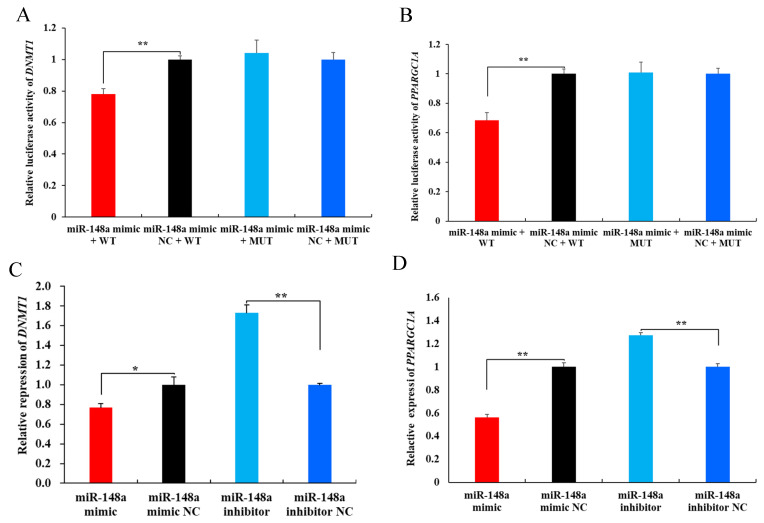
Validation of miR-148a with the predicted target genes *DNMT1* and *PPARGC1A*. (**A**,**B**) The luciferase activities of the target genes *DNMT1* and *PPARGC1A* for miR-148a detected using a dual luciferase reporter assay. (**C**,**D**) Effect of miR-148a on the expression levels of the target genes *DNMT1* and *PPARGC1A*. ** *p* < 0.01 and * *p* < 0.05.

Subsequent dual luciferase reporter assays were performed. In the HEK293T cells co-transfected with the miR-148a mimic and the wild-type pmiR-RB-Report™ vector, the luciferase activity of *DNMT1* decreased significantly to 77.92% of its NC group (*p* < 0.01). However, there was no significant difference in *DNMT1* luciferase activity between the miR-148a mimic and the miR-148a mimic NC in cells transfected with the mutant-type pmiR-RB-Report™ vector (Figure 7A, *p* > 0.05). Similarly, the miR-148a mimic decreased the luciferase activity of *PPARGC1A* in the wild-type vector (*p* < 0.01), but did not change the activity in the mutant-type vector (Figure 7B, *p* > 0.05). These results suggest that miR-148a targets *DNMT1* and *PPARGC1A* by binding to their 3’ UTR regions. 

RT-qPCR analysis further revealed that the miR-148a mimic reduced the expression of *DNMT1* and *PPARGC1A* in OMECs, while the miR-148a inhibitor increased their expression (Figure 7C,D, *p* < 0.01).

### 2.7. Expression Levels of the Target Genes in Ovine Mammary Gland Tissue during Different Development Periods

**Figure 8:**
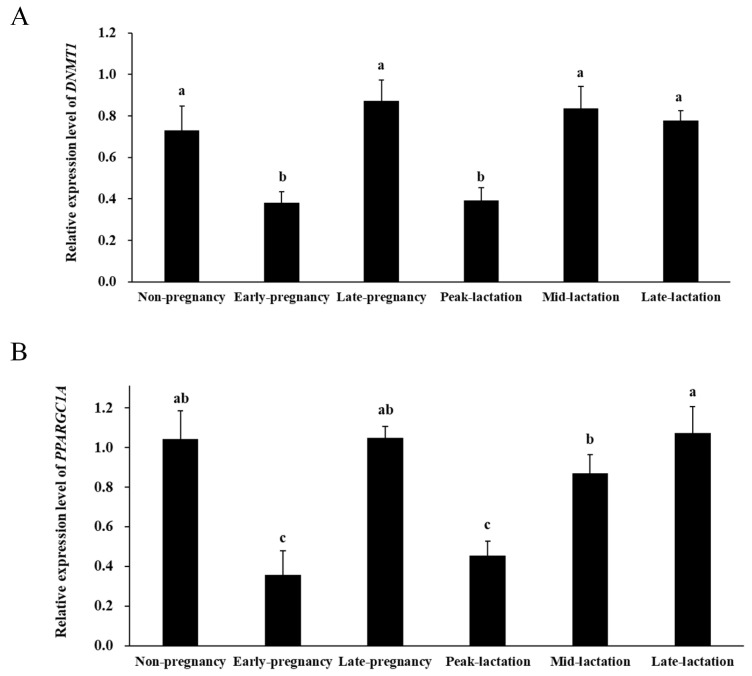
Expression levels of the target genes *DNMT1* (**A**) and *PPARGC1A* (**B**) for miR-148a in ovine mammary gland tissue during six different developmental periods. Values with different lowercase letters above the bars are different (*p* < 0.05).

In contrast to miR-148a, the expression levels of its target genes *DNMT1* and *PPARGC1A* were the lowest in ovine mammary gland tissue at peak lactation and early pregnancy, while their expression was higher in late lactation and non-pregnancy periods (Figure 8, *p* < 0.05). It was further found that miR-148 had a high negative correlation (|r| > 0.7) with the expression of *PPARGC1A* (r = −0.789, *p* < 0.001) in ovine mammary gland tissue, while it had a moderate negative correlation (0.3 < |r| ≤ 0.7) with the expression of *DNMT1* (r = −0.515, *p* = 0.029). These findings further demonstrate the negative regulatory effect of miR-148a on its target genes *DNMT1* and *PPARGC1A* in ovine mammary gland tissue.

## 3. Discussion

This study reported tissue- and temporal-specific expression patterns of miR-148a and its promoting effect on the viability, proliferation, and milk fat synthesis of OMECs in sheep. This study also verified the target relationship of miR-148a with *DNMT1* and *PPARGC1A*. In the study, miR-148a was found to be widely expressed in the eight ovine tissues investigated, with the highest expression levels in the mammary gland and liver, and the lowest levels in the *Longissimus dorsi* muscle and kidney. The result align with previous investigations in dairy goats [4] and sheep [10]. For example, miR-148a was expressed in the heart, liver, spleen, kidney, muscle, and mammary gland tissues of dairy goats, with the highest expression in the mammary gland [4]. In Tibetan sheep, miR-148a was most abundantly expressed in liver, followed by the spleen, adipose tissue, and heart, with the lowest levels in the lung, *Longissimus dorsi* muscle, and testis [10]. However, the previous study did not investigate the expression of miR-148a in the ovine mammary gland and ovary [10]. In our study, in addition to the mammary gland, the liver was one of the most abundant tissues for miR-148a expression. The liver is one of the most important organs for lipid metabolism, including triglyceride, cholesterol, and phospholipid. The high expression of miR-148a in the adult liver was found to be involved in regulation of the contents of triglyceride and cholesterol [11]. Taken together, these findings suggest that miR-148a may be involved in mammary gland development and milk fat synthesis in sheep. Among the mammary gland tissues investigated during six different developmental periods in this study, miR-148a showed the highest expression level at peak lactation and the lowest expression during the non-pregnancy period. The results were consistent with previous findings in mammary glands. For example, in a study by Chen et al. [12] investigating six different developmental periods of mammary gland tissue, miR-148a was expressed during all six periods, with the highest expression in early lactation and the least expression during non-pregnancy. The expression of miR-148a was 2.43-fold higher in lactating mammary glands than in non-pregnancy mammary glands in swamp buffalo [13]. Chen et al. [14] found that miR-148a was the most up-regulated miRNA in the ovine mammary gland during peak lactation. This likely reflects a temporal-specific expression pattern of miR-148a in mammary gland tissue. Given that the expression of miR-148a was the most abundant at peak lactation, a period associated with the highest average daily milk yield, it is therefore speculated that miR-148a expression may be positively correlated with milk yield. 

In the study, the effect of miR-148a on the proliferation of OMECs was comprehensively investigated by counting the number and percentage of Edu-labeled positive OMECs, analyzing the expression of the marker genes *CDK2* and *CDK4*, and observing its influence on the cell cycle. miR-148a was found to significantly increase the number and percentage of Edu-labeled positive OMECs. *CDK2* and *CDK4* are commonly used as proliferation marker genes to detect the proliferation state of MECs, preadipocytes, and ovarian granulosa cells [10]. The protein CDK2 promotes cell transition to the S phase by binding to Cyclin E, eventually increasing the rate of cell proliferation [15]. Similarly, CDK4 can combine with Cyclin D1 to form a kinase complex that promotes cell proliferation [16]. Our observation that miR-148 enhanced the expression of *CDK2* and *CDK4* indicates that the miRNA is favorable for OMECs’ proliferation. As cells in the S phase mainly replicate DNA, and the DNA content of the nucleus in this phase doubles, the number of cells in the S phase exhibits a positive correlation with the proliferation number of cells [10]. Our results indicated that miR-148a increased the proportion of OMECs in the S phase. Taken together, these findings suggest that miR-148a promotes the proliferation of OMECs. Meanwhile, the study also observed a promoting effect of miR-148a on the viability of OMECs. It has been reported that the proliferation number and viability of MECs are responsible for milk yield and milk quality in mammals [17]. It was therefore concluded that miR-148a contributed to lactation in sheep. The conclusion was supported by our findings, which showed that at peak lactation, miR-148a had a higher expression in the mammary gland of Small-tailed Han ewes with a higher milk yield and milk fat and milk protein contents. Muroya et al. [18] also found that the increased expression of miR-148a may be responsible for an increase in milk yield in dairy cows. In addition to OMECs, miR-148a was found to promote the proliferation ability of other cells, such as gastric cancer cells [19], glioblastoma cells [20], and multiple myeloma [21]. 

In the study, miR-148a not only increased the triglyceride content, but also enhanced the expression of three milk fat synthesis marker genes—*DGAT1*, *mTOR*, and *ABCG2*—in OMECs. The expression levels of these genes were found to be positively correlated with milk fat synthesis. *DGAT1* is considered one of the essential components in milk fat synthesis, as its encoded protein DGAT1 converts diglyceride to triglyceride by adding a third long-chain fatty acid [9]. The variant K231A in *DGAT1* has been associated with changes in milk fat and protein contents [22]. The protein encoded by *mTOR* is a phosphatidylinositol serine/threonine kinase and serves as a central hub in signaling pathways regulating milk fat and protein synthesis and the proliferation of MECs [23]. mTOR has been confirmed to promote milk fat synthesis and lipogenesis by activating its complex mTORC1 on sterol regulatory element binding protein 1 (SREBP-1) [24]. The protein ABCG2 is a member of the large ATP binding cassette family and is present in the milk fat globule membrane and apical membrane of the mammary alveolar epithelia [25]. Variation in *ABCG2* has been associated with the percentage and yield of milk fat [26]. Furthermore, *ABCG2* has been reported to be responsible for higher milk fat and protein contents and a higher milk fat yield [27]. Taken together, these findings suggest that miR-148a promotes milk fat synthesis in OMECs by up-regulating the expression of *DGAT1*, *mTOR,* and *ABCG2*. Our observation was supported by Chen et al. [12], who found that miR-148a promoted lipid droplet formation and increased the contents of triglyceride and cholesterol in goat MECs. Xia et al. [28] also found that miR-148a promoted triglyceride synthesis in bovine OMECs. The promoting effect of miR-148a on adipogenesis was also validated in ovine adipocytes [10] and 3T3-L1 cells [29]. However, negative regulation of miR-148a on triglycerides and cholesterol in bovine MECs has also been reported [11]. In this context, it was therefore suggested that the role of miR-148a in milk fat synthesis needs to be further investigated in more mammalian species. 

Upon the joint analysis of TargetScan7.2, miRDB, and miRWalk software, a total of 228 genes were predicted to target miR-148a. In our previous studies, the same ovine mammary gland tissues were used to investigate the expression profile of miRNAs and mRNAs [1,30]. Based on negative correlations of miRNAs in expression with mRNAs, predicted target genes were further screened. Finally, according to the functions of the screened genes in the mammary gland reported in the previous literature [12,31,32], *DNMT1* and *PPARGC1A* were chosen to validate their target relationship with miR-148a. In a subsequent dual luciferase reporter assay, these two genes were confirmed as target genes of miR-148a. The negative correlation of miR-148a with the expression levels of these two target genes in ovine mammary gland tissue further suggests that miR-148a may be involved in regulating ovine mammary gland development and lactation performance by targeting *DNMT1* and *PPARGC1A*. 

*DNMT1* has been identified as a target gene of miR-148a in human bone mesenchymal stromal cells [33] and breast cancer [34]. DNA methylation is the best-studied epigenetic mechanism catalyzed by enzymes in the DNA methyltransferase (DNMT) family, which includes DNMT1, DNMT3a, and DNMT3b. Of these members, DNMT3a and DNMT3b are responsible for the de novo methylation of unmodified DNA, whereas DNMT1 maintains DNA methylation during DNA replication [35]. When DNMT1 is located at the promoter region of a gene, methylation usually represses gene expression. During mammary gland development and milk synthesis, numerous genes involved in the viability, proliferation, and milk synthesis processes of MECs are susceptible to methylation. Previous studies have shown that miRNAs regulate lactation performance and mammary gland development by influencing DNA methylation via targeting the 3’ UTR of *DNMT1* [3]. These suggest that miR-148a affects the expression of important functional genes by modulating the methylation of DNMT1 protein, which in turn influences the vitality, proliferation, and milk synthesis of OMECs. For example, DNMT1-mediated DNA methylation of the lactation-related genes *STAT5* and *PPARγ*, and a milk-fat-related gene *Lats2*, regulates milk synthesis and the activity of MECs [31,36]. Szyf et al. [37] found that *DNMT1* interacted with the proliferation-related genes *PCNA*, *p21,* and *p16* to affect the cell cycle, suggesting *DNMT1* may regulate OMECs’ viability and proliferation. In addition, Wang et al. [31] found that *DNMT1* expression was negatively correlated with milk yield, milk protein, and milk fat in dairy cows. Given that miR-148a was up-regulated in the mammary gland tissues of a sheep breed producing a higher milk yield, milk fat, and milk protein in this study, it was concluded that miR-148a promoted lactation and milk protein and fat synthesis by down-regulating *DNMT1* expression in sheep. The target relationship of miR-148a with *PPARGC1A* was also confirmed in MECs of dairy goats using a dual luciferase reporter assay [4]. They found that miR-148a increased triglyceride synthesis by down-regulating *PPARGC1A* expression, consistent with our results. PPARγ is a key regulator of adipocyte differentiation as it regulates the expression of genes related to fatty acid, while PPARGC1A regulates mammary gland metabolism, milk yield, and milk protein by interacting with PPARγ [38]. Moreover, *PPARGC1A* mainly affects long-chain unsaturated fatty acids, medium-chain saturated fatty acids, and milk fatty acid composition [32]. Taken together, these findings suggest that miR-148a regulates lactation performance and milk fat synthesis by targeting *DNMT1* and *PPARGC1A*.

Previous studies have shown that a single miRNA can target the 3’ UTR of either a single mRNA or multiple different mRNAs [39]. This phenomenon has been confirmed for miR-148a. In addition to *DNMT1* and *PPARGC1A*, miR-148a has been found to target bone morphogenetic protein 7 (BMP7) to reduce its expression [40]. The target relationships of miR-148a with rho-associated coiled-coil protein kinase-1 (ROCK1) in C2C12 myoblast [41] and *PTEN* in ovine preadipocytes [10] have also been confirmed. The complex relationships of miR-148a with these target genes suggest that miR-148a may play pleiotropic roles in multiple cellular processes and indicate that miR-148a may be involved in mammary gland development and regulation of milk components by targeting other functional genes. However, the speculation will need further investigation in future.

In addition to miRNAs, sex hormones and various growth factors contribute to the growth and development of the mammary gland and lactation process, including estrogen, progesterone, glucocorticoids, growth hormone, prolactin, thyroid and adrenal hormone, etc. To better explain the molecular mechanism underlying ovine mammary gland development and lactation, we will further investigate the effect of hormones on the mammary gland and milk synthesis in future research. 

## 4. Materials and Methods

### 4.1. Ovine Tissue Sample Collection

All animal experiments, including the collection of ovine tissue samples in this study, were approved by the Animal Experiment Ethics Committee of Gansu Agricultural University, Lanzhou, China (approval number GSAU-ETH-AST-2021-027). 

Three independent experiments were conducted to collect ovine tissue samples and the ewes used in each experiment were different. The details are presented below:

First, six healthy Small-tailed Han ewes were selected from the Kangyuan Sheep Breeding Company (Huining County, Baiyin, China). The ewes all produced triplet lambs and were in their fourth parity. Their mammary gland biopsy tissue samples were collected using a surgical biopsy during six physiological periods, including non-pregnancy (25 d after the cessation of lactation), early pregnancy (35 d of gestation), late pregnancy (135 d of gestation), peak lactation (22 d postpartum), mid-lactation (36 d postpartum) and late lactation (67 d postpartum). The tissues were used to culture OMECs and analyze the expression of miR-148a and its target genes in the mammary gland during different physiological periods.

Second, at peak lactation (22 d postpartum), six separate healthy Small-tailed Han ewes were selected at the Kangyuan Sheep Breeding Company. The age, parity, and lambing number of the ewes were the same. They were slaughtered to collect eight tissues, including the mammary gland, *Longissimus dorsi* muscle, heart, kidney, spleen, lung, ovary, and liver. The tissues were used to compare the expression levels of miR-148a in different tissues.

Third, under the same of feeding condition, age, parity, and lambing number, six healthy Small-tailed Han ewes and six healthy Gansu Alpine Merino ewes were selected. Their milk samples were collected in a 50 mL milk collecting cup, and the milk fat content and milk protein were tested using a milk composition analyzer (UC, Hangzhou, China). The average daily milk yield within 30 days postpartum and the milk fat content and milk protein content of the Small-tailed Han ewes were 1357 g, 7.42%, and 5.37%, respectively, while the measurements for the Gansu Alpine Merino ewes were 853 g, 5.66%, and 4.67%, respectively [42]. At peak lactation (22 d postpartum), their mammary gland tissue samples were collected using surgical biopsy. The mammary gland samples were used to compare the difference in the expression of miR-148a between the two sheep breeds.

### 4.2. Tissue Expression of miR-148a and Its Target Genes

Total RNA from the tissues described above was isolated using Trizol reagent (Invitrogen, Carlsbad, CA, USA). The concentration and quality of the RNA were checked using a NanoDrop 8000 spectrophotometer (NanoDrop Technologies, Woltham, MA, USA). Super Script TM II reverse transcriptase (Invitrogen, Carlsbad, CA, USA) was used to produce cDNA of qualified RNA. PCR primers were designed for detection of the expression of miR-148a and its target genes (Table 1), and *U6* and *β-actin* were chosen as internal reference genes for miR-148a and mRNAs, respectively [8]. The RT-qPCR was carried out in triplicate using the 2 × ChamQ SYBR qPCR Master Mix (Vazyme, Nanjing, China). The relative expression levels of miR-148a and its target genes in tissues were calculated using the 2^−ΔΔCT^ method [43].

### 4.3. Culture and Purification of OMECs

Based on our previously established method [8], cells isolated from the ovine mammary gland were cultured in 24-well plates with a basal DMEM/F12 growth medium (Invitrogen, CA, USA), including 10% fetal bovine serum, 10 ng/mL epidermal growth factor 1, 5 μL/mL insulin-transferrin-sodium selenium, and 5 μg/mL hydrocortisone. Subsequently, purified OMECs were obtained by removing fibroblasts from raw cells using 0.05% trypsin–EDTA (Invitrogen, CA, USA) for 2 minutes. The purity of OMECs was identified using cytokeratin 18 and vimentin immunofluorescence staining analysis. At the beginning of the experiment, 2 μg/mL prolactin (USBiological, Salem, MA, USA) was added to the DMEM/F12 medium to induce the lactation of OMECs.

### 4.4. Cell Transfection and CCK8 Assay

A miR-148a mimic, a miR-148a inhibitor, and their negative controls (named miR-148a mimic NC and miR-148a inhibitor NC) were synthesized by RiboBio Co., Ltd. (Guangzhou, China), and they were then transfected into OMECs cultured in 24-well plates in triplicate using INVI DNA & RNA Transfection Reagent™ (Invigentech, Irvine, CA, USA), when the confluence of the cells reached 70% to 80%. Subsequently, after transfection for 46 hours, 30 μL CCK8 reagent (Vazyme, Nanjing, China) was added to each well, and the culture was continued for 2 hours. The absorbance of OMECs at 450 nm was measured using a Thermo Scientific Microplate Reader (Thermo Scientific, MA, USA). For each group of OMECs transfected, five wells were randomly selected to assess the cell viability. 

### 4.5. Effect of miR-148a on the Proliferation of OMECs

After transfection of the miR-148a mimic and miR-148 inhibitor into OMECs for 44 hours, the following three experiments were performed to investigate the effect of miR-148a on the proliferation of OMECs. (1) A total of 100 mL of 50 nM Cell-Light ™ Edu solution (Beyotime, Shanghai, China) was added to each well and then cultured for 4 hours. The Edu staining result of OMECs was determined using a microscope IX73 (Olympus, Tokyo, Japan), and the proportion of Edu-labeled positive OMECs was counted by Image-Pro Plus V7.0. (2) OMECs were treated using a cell cycle analysis kit (Thermo Fisher Scientific, Waltham, MA, USA), and the content of DNA in each phase was measured using a AccuriC6 flow cytometry system (BD Biosciences, New Jersey, NJ, USA). (3) Total RNA was extracted from four groups of transfected OMECs using Trizol reagent (Invitrogen, CA, USA). The expression levels of two cell proliferation marker genes *CDK2* and *CDK4* were detected using RT-qPCR analysis. Ovine *β-actin* and *GAPDH* were chosen as internal references [17], and their primers are listed in Table 1.

### 4.6. Effect of miR-148a on the Milk Fat Synthesis of OMECs 

After transfection of the miR-148a mimic, miR-148a inhibitor, and their NC group into OMECs for 48 hours, the expression levels of three milk fat synthesis marker genes were quantified using the method described above. They included *mTOR*, *DGAT1*, and *ABCG2*. Meanwhile, 300 uL of lysate solution was added to OMECs and then centrifugated to collect the supernatant. The concentration of triglycerides in OMECs was determined in quadruplicate using a triglyceride content assay kit (Solarbio, Beijing, China).

### 4.7. Dual Luciferase Reporter Assay

TargetScan7.2, miRDB, and miRWalk were used to predict the target genes of miR-148a, and the results from these three software were compared. Based on the roles of predicted genes in lactation and milk ingredients, *DNMT1* and *PPARGC1A* were chosen to perform a dual luciferase reporter assay to verify their target relationship with miR-148a. Briefly, the 3’ UTR sequences of the two genes binding with miR-148a were amplified using PCR primers (Table 1). Amplified product was ligated to a pmiR-RB-Report™ vector to produce a wild-type pmiR-RB-Report™ vector. Meanwhile, the target sequences of the 3’ UTR regions of the target genes for miR-148a were mutated to their complementarily sequences and then ligated to a vector to form a mutant-type pmiR-RB-Report™ vector. The vectors constructed were confirmed by Sanger sequencing.

A total of 20 pmol miR-148a mimic and 0.20 µg plasmid extracted from the wild-type vector were co-transfected into the HEK293 T cells cultured in DMEM/F12 medium containing 10% PBS and 1% penicillin–streptomycin using INVI DNA & RNA Transfection Reagent™ (Invigentech, CA, USA). Three control groups were also transfected into HEK293 T cells cultured in 24-well plates, including miR-148a mimic NC + wild-type vector, miR-148a mimic + mutant-type vector, and miR-148a mimic NC + mutant-type vector. After transfection for 48 hours, the luciferase activities of *DNMT1* and *PPARGC1A* were detected using a dual luciferase reporter kit (Promega, Madison, WI, USA) on a Varioskan LUX Microplate Reader (Thermo Lifetech, Waltham, MA, USA).

### 4.8. Statistical Analysis

The data were analyzed using SPSS 22.0 (IBM, New York, NY, USA). A two-tailed Student′s t-test was used to compare the differences between two groups, while a one-way ANOVA was used to analyze difference between multiple groups (e.g., the expression levels of miR-148a and mRNAs in different tissues and periods). Pearson correlation coefficients were calculated on the expression levels of ovine mammary gland tissue during six different developmental periods between miR-148a and its target genes. The value of the correlation coefficient r was used to estimate the degree of correlation. Correlations with |r| ≤ 0.3, 0.3 < |r| ≤ 0.7, and |r| > 0.7 were defined as weak correlation, moderate correlation, and high correlation, respectively. 

## 5. Conclusions

Our results indicate that the expression of miR-148a exhibits obvious tissue- and temporal-specific patterns, with the highest expression level in the mammary gland among the eight tissues investigated and during the peak lactation period among the six mammary gland developmental periods. Meanwhile, miR-148a improves the viability, proliferation, and milk fat synthesis of OMECs in sheep by targeting *DNMT1* and *PPARGC1A*. This study contributes to a deep understanding of the effect of miR-148a on mammary gland development, and it also provides a technology route for improving milk production performance including the milk fat level by regulating the expression level of miR-148a in sheep.

## Figures and Tables

**Table 1:** PCR primers used in the study.

Name	Forward (5’→3’)	Reverse (5’→3’)	Purpose of Primers
*DNMT1* (WT) ^1^	CCGCTCGAGCCTATCACCCATGTTTCTG	GAATGCGGCCGCGTCTTAATTTCCACTTCA	Construction of pmiR-RB-Report™ vector
*PPARGC1A* (WT) ^1^	CCGCTCGAGGAGCACGGTGTTACATTA	GAATGCGGCCGCGGGCTAGGAGGTTTATTG	
*DNMT1* (MUT) ^2^	CAGCGACGTGACATATTGTTGTATTTTCACATGTCAATCG	AATATGTCAGTCGCTGGGGATCTCTGGTGG	
*PPARGC1A* (MUT) ^2^	GTTACAGCTAACGTGACTAAATGCAGCCTTCTTT	GTCACGTTAGCTGTAACAAAAAAAGAACATCATTG	
miRNA-148a	TCAGTGCACTACAGAACTTTGT	TGGTGTCGTGGAGTCG	RT-qPCR
*U6*	GGAACGATACAGAGAAGATTAGC	TGGAACGCTTCACGAATTTGCG	
*CDK2*	AGAAGTGGTGGCGCTTAAAA	TCTTGAGATCCTGGTGCAGA	
*CDK4*	GCTTTTGAGCATCCCAATGT	AGGTCTTGGTCCACATGCTC	
*GAPDH*	CACAGTCAAGGCAGAGAACG	CAGCCTTCTCCATGGTAGTG	
*β-actin*	AGCCTTCCTTCCTGGGCATGGA	GGACAGCACCGTGTTGGCGTAGA	
*mTOR*	TGCTAACTACCTTCGGAACCT	TGAAAGTGTCCCCTGCCAT	
*DGAT1*	CCACCATTCTCTGCTTCCCA	AGCTTGAGGAAGAGGATGGT	
*ABCG2*	AGTTACGAGGTTTCCATCCCA	CGACAAAGTAGAAAGCCAGTCT	
*DNMT1*	TTCTCACTGCCTGACGATGT	TGACTTTAGCCAGGTAGCCC	
*PPARGC1A*	GACTCAAGTGGTGCAGTGAC	GGCAATCCGTCTTCATCCAC	

^1^ The primers were used to amplify the 3’ untranslated regions (3’ UTR) of the target genes when a wild-type (WT) pmiR-RB-Report™ vector was constructed. ^2^ The primers were used to amplify mutant sequences of the 3’ UTR of the target genes bound with miR-148a when a mutant-type (MUT) pmiR-RB-Report™ vector was constructed.

## Data Availability

The data presented in this study are available in the article.
